# Divergent Population Genetic Structure of the Endangered *Helianthemum* (Cistaceae) and Its Implication to Conservation in Northwestern China

**DOI:** 10.3389/fpls.2016.02010

**Published:** 2017-01-05

**Authors:** Zhihao Su, Bryce A. Richardson, Li Zhuo, Xiaolong Jiang

**Affiliations:** ^1^Key Laboratory of Biogeography and Bioresource in Arid Land, Xinjiang Institute of Ecology and Geography, Chinese Academy of SciencesUrumqi, China; ^2^USDA Forest Service, Rocky Mountain Research StationProvo, UT, USA; ^3^Library, Xinjiang Normal UniversityUrumqi, China; ^4^Shanghai Chenshan Plant Science Research Center, Shanghai Chenshan Botanical Garden, Chinese Academy of SciencesShanghai, China

**Keywords:** *Helianthemum*, Yili Valley, western Ordos Plateau, genetic diversity, genetic structure, conservation implication

## Abstract

Population genetic studies provide a foundation for conservation planning, especially for endangered species. Three chloroplast SSRs (*mtrnSf-trnGr, mtrnL2-trnF*, and *mtrnL5-trnL3*) and the internal transcribed spacer were used to examine the population structure of *Helianthemum* in northwestern China. A total of 15 populations of the genus were collected. Nine chloroplast haplotypes and two nuclear genotypes were detected. Both the nuclear and chloroplast data showed two lineages in *Helianthemum songaricum*, respectively, distributed in Yili Valley and western Ordos Plateau. A total of 66.81% (*p* < 0.001) of the genetic variation was supported by this lineage split. A Mantel test showed a significant correlation between genetic distance and geographical distance (*r* = 0.937, *p* < 0.001). Based on genetic analyses, cpSSRs data support strong genetic divergence between regions. We speculate that the climate change during the late Tertiary and early Quaternary isolated *H. songaricum* into their current distribution, resulting in interruption of gene flow, leading to isolation and genetic divergence between the two regions. Meanwhile, possible selfing would increase genetic drift in small fragmented populations, that might account for the observed genetic divergence in both regions. Given the loss of genetic diversity and genetic divergence in small populations of *Helianthemum* in northwestern China immediate conservation management steps should be taken on the species.

## Introduction

*Helianthemum* is a shrub or subshrub mostly distributed in the Mediterranean, extending to Central Asia (Yang and Michael, 2007). *Helianthemum songaricum* and *H*. *ordosicum* in northwestern China is disjunctively distributed in Yili Valley of Xinjiang and western Ordos Plateau of Inner Mongolia, growing in rocky hills and slopes in steppe-desert regions between 1000 and 1400 m. It has spine-tipped branches, stipulate leaves, yellow flowers, and insect pollinated. Because of anthropogenic activities, such as grazing, mining, and the heavy harvest of firewood over the last few decades, *H. songaricum* and *H*. *ordosicum* has been in decline and become highly fragmented. As a result, it was listed as endangered in the China Species Red List (Fu, 1992).

There is disagreement with the taxonomic status of species in *Helianthemum* in northwestern China. Initially, taxonomists considered only one species of *Helianthemum* in northwestern China, *Helianthemum songaricum* Schrenk (Li, 1990). However, later studies found that there was a significant difference of pollen morphology and chromosome number between populations in Yili Valley and those in western Ordos Plateau. In the Yili Valley pollen was striate with a cytotype of 2n = 20, and in the western Ordos Plateau the pollen was perforate with 2n = 40 (Mo et al., 1997; Cao et al., 2000). Based on these results, a new taxon, *H. ordosicum*, was proposed in western Ordos (Zhao et al., 2000). More recently, Yang and Michael (2007) recognized only one species, *H. songaricum*. In our previous phylogeographic study, two chloroplast intergenic spacers data supported two species: *H*. *songaricum* and *H. ordosicum*. (Su et al., 2011).

Correctly defining the taxonomy and populations (i.e., intraspecific manage units, MU) is essential to make effective conservation strategies for endangered species (Frankham et al., 2002). Incorrect taxonomy would lead to ill-conceived management strategies. For example, outcrossing of different taxa can create inviable or infertile offspring (Barton and Hewitt, 1981; Coyne and Orr, 1989) and dismantle of coadapted complexes (Mayr, 1963; Shields, 1982; Templeton, 1987), leading to outbreeding depression. Outcrossing depression can also be observed in crosses at the intraspecific level (Geiger, 1988; Waser and Price, 1989). Populations that have adapted to different habitats could also suffer from outbreeding depression and should treated as a distinct manage unit to avoid outcrossing (Frankham et al., 2002).

Information on genetic structure can resolve ambiguous classifications and establish a foundation for conservation genetic planning. Chloroplast simple sequence repeats (cpSSRs) are a highly polymorphic molecular tool in the population genetic analysis (Vendramin et al., 1996; Morgante et al., 1997; Ebert and Peakall, 2009). Besides their high mutation rates, they have other specific features. Because of their uniparental inheritance, they could show pronounced level of population differentiation (Ennos, 1994; Vendramin et al., 1999; Flannery et al., 2006). In addition, in monoecious species, uniparentally inherited genomes have only half the effective population size (Birky, 1988), therefore, these genomes are sensitive to historical bottlenecks (Morgante et al., 1997). Though the apparent advantages, chloroplast DNA markers might only provide partly genetic information of a species (Mäder et al., 2010), and combination with biparentally inherited nuclear DNA markers would present a more integral view of population structure and demography history (Burban and Petit, 2003; Petit et al., 2005).

The primary conclusion in our previous study, that was significant genetic divergence existed between Yili Valley and western Ordos Plateau, was only based on two chloroplast spacers (trn*D*-trn*T* and rps16-trn*K*) and need to be further improved with nuclear genome data. In addition, population structure in the two regions are still unclear because of the limited polymorphism in the two chloroplast spacers (Su et al., 2011). Here, we use three highly polymorphic cpSSRs and nuclear Internal Transcribed Spacer (ITS) sequence to investigate the full genetic structure of *Helianthemum* in northwestern China to address the following questions: (1) Whether populations of *Helianthemum* in western Ordos Plateau represent a distinct taxa, *H. ordosicum*? (2) if so, what is the genetic structure within the two species? (3) What are the conservation implications from the genetic structure analysis?

## Materials and methods

### Sampling

**Figure 1:**
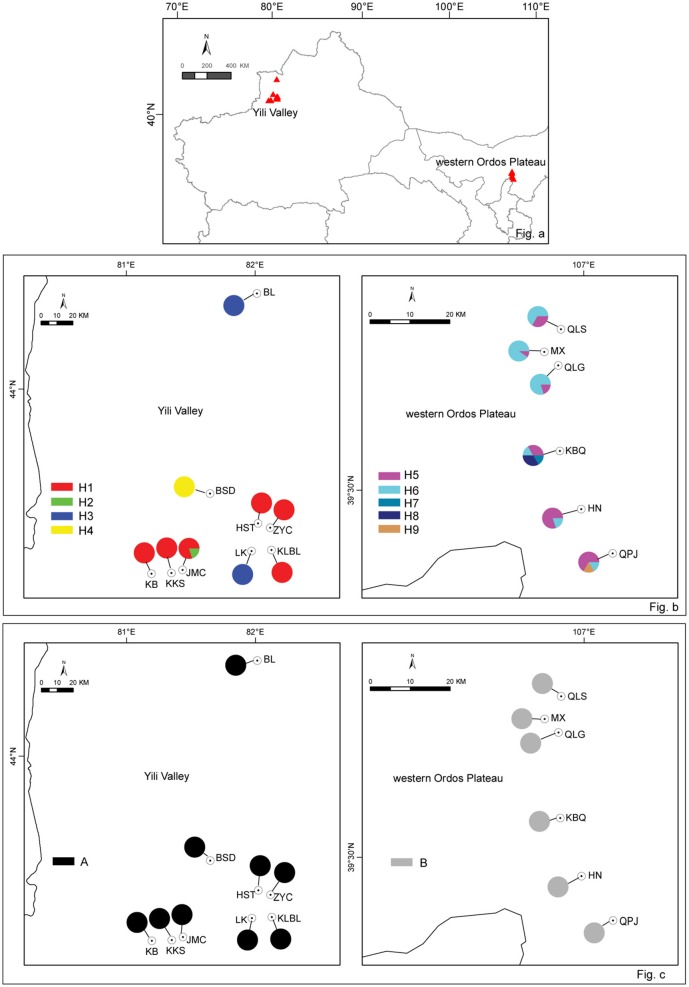
**Sampling distribution of ***Helianthemum*** in China (a)**, the cp haplotype distribution **(b)**, and ITS genotypes distribution in *Helianthemum* **(c)**. Population numbers correspond to those in Table 1; cp haplotypes to those in Table 2, pie-charts represent haplotype frequency.

In 2010 and 2014, *H. songaricum* and *H*. *ordosicum* was sampled throughout its distribution in northwestern China. A total of 15 populations of the species were collected: nine from Yili Valley and six from western Ordos Plateau (Figure 1A). The geographical locations of the collection sites are presented in Table 1. Six to twelve individuals were sampled in each population. Fresh leaves were dried in silica gel and stored at 4°C until DNA extraction.

**Table 1:** **Details of sample locations, sample size, and genetic variation for 15 populations of ***Helianthemum*****.

**Region**	**Number**	**Location**	**Code**	**Latitude (N)**	**Longitude (E)**	**Altitude (m)**	**Sample Number**	***Cp* Haplotype**	**ITS Genotype**
Yili Valley	1	Junmachang	JMC	43.23	81.98	1318	10	8H1,2H2	8A
	2	Heishantou	HST	43.58	82.47	810	12	12H1	8A
	3	Kekesu	KKS	43.2	81.93	1166	10	10H1	8A
	4	Zhongyangchang	ZYC	43.57	82.57	952	12	12H1	8A
	5	Kebo	KB	43.17	81.75	1246	10	10H1	8A
	6	Bole	BL	44.83	82.05	566	8	8H3	8A
	7	Longkou	LK	43.42	82.47	855	10	10H3	8A
	8	Kalabula	KLBL	43.45	82.62	829	10	10H1	8A
	9	Baishidun	BSD	43.68	82.05	1220	10	10H4	8A
Western Ordos Plateau	10	Mengxi	MX	39.8	106.88	1260	10	1H5,9H6	7B
	11	Qianligou	QLG	39.77	106.92	1450	10	2H5,8H6	7B
	12	Kabuqi	KBQ	39.58	106.92	1248	12	4H5,2H6,2H7,4H8	8B
	13	Qipanjing	QPJ	39.35	107.07	1328	12	8H5,2H6,2H9	8B
	14	Qianlishan	QLS	39.85	106.93	1359	6	2H5,4H6	6B
	15	Hainan	HN	39.45	106.98	1230	10	8H5,2H6	8B

*Figures before the genetic variation represent the number of the variation in the population*.

### DNA extraction, CpSSRS, and ITS sequencing

Total genomic DNA was extracted from dried leaves by the CTAB method (Doyle and Doyle, 1987). Polymerase chain reactions (PCR) were carried out in a volume of 25 μL reaction mixtures containing 4 mM MgCl_2_, 0.2 mM dNTP, 0.5 μmol primer, and 1 U Taq polymerase (Applied Biosystems, Foster City, Calif.), implemented in a Biorad T100 thermocycler (Biorad). The cpSSRs were amplified using three *Helianthemum*-specific polymorphic loci detected in regions *trnL-trnF, trnL5-trnL3*, and *trnS-trnG*: *mtrnSf-trnGr, mtrnL2-trnF*, and *mtrnL5-trnL3* (see Soubani et al., 2014), and following the temperature profile: 95°C for 4 min; 30 cycles of 92°C for 45 s; 57°C for 45 s; and 72°C for 1 min; linked a extension at 72°C for 10 min; ITS2 region was amplified using primers of Sun et al. (1994), and following the temperature profile: 94°C for 5 min; 35 cycles of 94°C for 30 s; 52°C for 45 s; and 72°C for 1 min; linked a extension at 72°C for 8 min. The cpSSRs products were separated by capillary electrophoresis, with an ABI 3730xl (Applied Biosystems) automated sequencer. CpSSRs fragment sizes were determined in Geneious version 7.0 using the package Plugin (Kearse et al., 2012), using Gene-flo 625 (Chimerx) as the internal lane standard. ITS2 amplified primers were used in sequencing reactions conducting in the DYEnamic ET Terminator Kit (Amersham Pharmacia Biotech). Sequencing were carried out in ABI 3730xl. ITS2 electropherograms were edited and assembled in SEQUENCHER 4.8 (Gene Codes, Ann Arbor, MI, USA), then the sequences were aligned in CLUSTALW (Thompson et al., 1994), and refined by visual inspection.

### Population genetic analysis

For ease of presentation in this study, the terms “locus” refers to a cpSSR site, and “allele” refers to a length-variant at a cpSSR site. Alleles of the three plastid loci were scored, respectively, treated as ordered characters and then combined together as multilocus haplotypes, assuming a stepwise pattern in mutation (Ohta and Kimura, 1973). Using stirling probability distribution and Bayes's theorem, the completeness of haplotype sampling in this study was estimated (Dixon, 2006).

The number of different alleles (Na), the effective number of alleles (Ne), and Nei's genetic distence (Nei, 1978), were caculated in GenAlEx 6.5 software (Peakall and Smouse, 2006). Within-population diversity (h_S_), total gene diversity (h_T_), genetic differentiation index (G_ST_, leaves out mutation steps between haplotypes; N_ST_, includes mutation steps between haplotypes) were calculated in the program HAPLONST, using U-test to determine whether N_ST_ is significantly larger than G_ST_.

Using pairwise population differentiation measures (F_ST_) as the variance components (Wright, 1965), analysis of molecular variance (AMOVA) was performed to study the partition of total genetic variation within and among populations, conducted in ARLEQUIN v.3.01 (Excoffier et al., 1992). The significance test used 10,000 permutations. To evaluate the population genetic structure, a Neighbor-Joining network (NJ) of the 15 populations was constructed in MEGA 6.0 (Tamura et al., 2013), using Nei's genetic distance matrix. This genetic distance matrix was also used to perform principal coordinate (PCO) analysis in GenAlEx 6.5 (Peakall and Smouse, 2006). To reveal the genetic divergence between the two regions, a Mantel test was performed in ARLEQUIN v.3.01, with 10,000 permutations significance test. Geographical distance was calculated in GEODIS 2.5 (Posada et al., 2000), natural-log transformed in Excel 2000, and then correlated with the Nei's genetic distances.

### Phylogenetic analysis

To analyse the genealogical relationships among all the chloroplast haplotypes, a network was constructed using median-joining method conducted in NETWORK v. 4.600 (Bandelt et al., 1999).

## Results

### Allele and sequence analysis

A total of 13 alleles were detected in the three cpSSRs: three alleles in *mtrnSf-trnGr*, four alleles in *mtrnL2-trnF*, and six alleles in *mtrnL5-trnL3*. The 154 individuals sampled from 15 populations yielded 9 haplotypes (Table 2). Using the method described in Dixon (2006), the estimated probability of haplotype completeness was 1.0, suggesting that we have sampled almost all potential haplotypes in this study. For the ITS2 region, the aligned sequence length was 449 bp, and one informative nucleotide substitution (G/T) was found in position 173. Two nuclear genotypes (A and B) were identified in 116 individuals from 15 populations. GenBank accession numbers of the ITS2 sequences are KY314618-KY314619.

**Table 2:** **Nine haplotypes of ***Helianthemum*** recognized on basis of three chloroplast SSRs, ***mtrnSf-trnGr, mtrnL2-trnF***, and ***mtrnL5-trnL3*****.

**Haplotypes**	***mtrnSf-trnGr***	***mtrnL2-trnF***	***mtrnL5-trnL3***
H1	133	224	239
H2	133	219	239
H3	133	224	238
H4	133	224	240
H5	133	223	243
H6	133	223	244
H7	135	222	243
H8	134	223	244
H9	135	223	242

### Haplotype patterns

The cpSSR haplotypes were partitioned among the two regions: Yili Valley and western Ordos Plateau. Haplotypes H1-H4 were distributed in Yili Valley, and haplotypes H5–H9 were distributed in western Ordos Plateau. Between the two regions, there was no shared haplotypes (Figure 1B). In Yili Valley, haplotype H1 was widespread in six populations of the total nine populations; rare haplotype H2 was found in population JMC; haplotype H3 was isolated in populations BL and LK, and haplotype H4 was isolated in population BSD. In western Ordos Plateau, haplotypes H5 and H6 were found in each population; rare haplotypes, H7 and H8 were found in population KBQ, and H9 was found in population QPJ (Figure 1B). For ITS genotype, all the individuals in Yili Valley contained one genotype (A), and all the individuals in western Ordos Plateau contained the other genotype (B) (Figure 1C).

**Figure 2:**
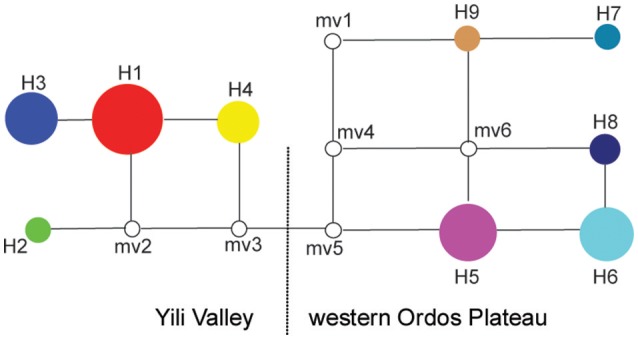
**Median-joining network of ***Helianthemum*** haplotypes**. The blank circles indicate missing or inferred haplotypes; the circle size is proportional to haplotype frequency; haplotypes in the network showed in the same colors correspond to those in the geographical distribution, Figure 1B.

The genetic relationships among the nine haplotypes also supports the disjunct geography of the Yili Valley and Ordos Plateau. The haplotypes found in the two geographic regions are also distinct based on the haplotype network (Figure 2). These two regional lineages, haplotypes H1-H4 corresponding to Yili Valley and H5-H9 corresponding to western Ordos Plateau, were connected by at least two hypothetical haplotypes (mv3 and mv5).

### Genetic diversity and genetic structure

**Figure 3:**
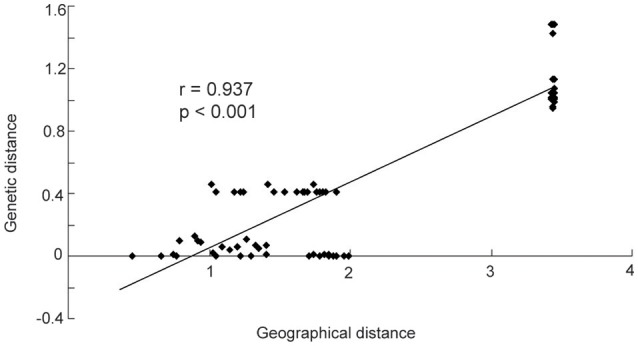
**The figure shows a significant relationship between geographic and genetic distance (***r*** = 0.937, ***p*** < 0.001)**.

The mean different alleles number (Na) was 1.267, and effective alleles number (Ne) was 1.150. Across the entire study area, total gene diversity (h_T_) was 0.805 (SE 0.0632), and within-population gene diversity (h_*S*_) was 0.209 (SE 0.0679). Genetic differentiation index G_ST_ was 0.740 (SE 0.0784), and N_ST_ was 0.803 (SE 0.0617). As shown by the results of a *U*-test (*U* = 0.99, *p* < 0.01), N_ST_ was significantly higher than G_ST_, suggesting a significant phylogeographical structure within *Helianthemum*. In Yili Valley, h_T_ was 0.583 (SE 0.1501), h_S_ was 0.04 (SE 0.0395), and genetic differentiation index was (G_ST_ = 0.932, N_ST_ = 0.934); in western Ordos Plateau, h_T_ was 0.634 (SE 0.0748), h_S_ was 0.463 (SE 0.0836), and genetic differentiation index was (G_ST_ = 0.270, N_ST_ = 0.226). AMOVA analysis showed that 79.32% (*p* < 0.001) of the total variation occurred among populations. When populations were grouped by geographical region, 66.81% (*p* < 0.001) of the total variation occurred among the regions (Table 3). In Yili Valley, 93.22% (*p* < 0.001) of the total variation occurred among populations; in western Ordos Plateau, 22.36% (*p* < 0.001) of the total variation occurred among populations. Mantel's test showed a significant correlation between genetic distance and geographical distance (*r* = 0.937, *p* < 0.001, Figure 3).

**Table 3:** **Results of analysis of molecular variance for ***Helianthemum*** based on chloroplast SSRs data**.

**Source of variation**	***d.f.***	**Sum of squares**	**Variance components**	**Percentage of variation (%)**
Among populations	14	81.473	0.5608	79.32^*^
Within populations	137	20.033	0.1462	20.68
Yili Valley vs. western Ordos Plateau				
Among geographic regions	1	53.099	0.7002	66.81^*^
Among populations within regions	13	28.375	0.2017	19.24^*^
Within populations	137	20.033	0.1462	13.95^*^
Yili Valley				
Among populations	8	21.791	0.2650	93.22
Within populations	83	1.600	0.0192	6.78
western Ordos Plateau				
Among populations	5	6.583	0.0983	22.36
Within populations	54	18.433	0.3414	77.64

* *P < 0.001*.

**Figure 4:**
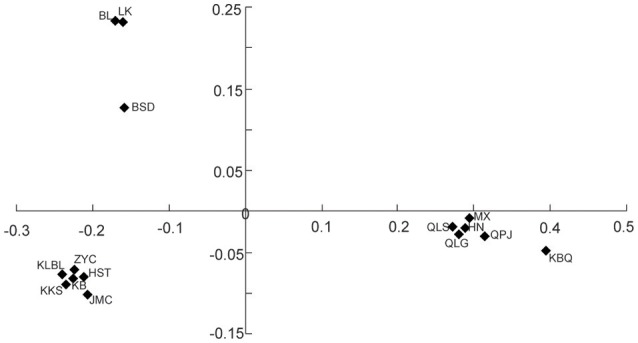
**Plots of the first two coordinates based on pairwise population differentiation (Nei' s) matrix of ***Helianthemum*****.

**Figure 5:**
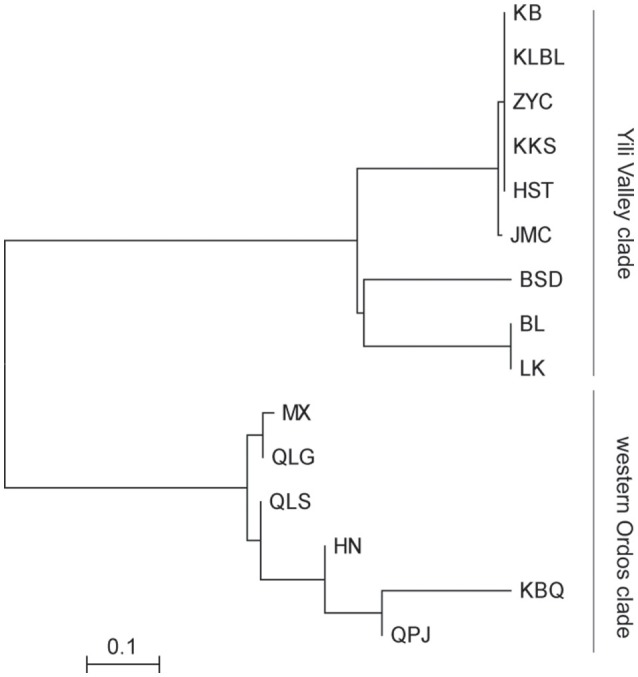
**Neighbor-Joining tree of the 15 ***Helianthemum*** populations constructed using Nei's genetic distance matrix**.

The PCO plot illustrates the distinct differences between regions and differences within regions. The first two axis accounted for 76.14 and 13.66% of the total variation, respectively (Figure 4). The first axis separated all the *Helianthemum* populations into two groups, one including populations in Yili Valley and the other including populations in western Ordos Plateau. The second axis separated all the populations in Yili Valley into three groups, one including population BSD, one including populations BL and LK, and another included the remaining populations. The PCO plots suggested a high genetic divergence between Yili Valley and western Ordos Plateau population, and also a high genetic divergence among populations within Yili Valley. The PCO plot was consistent with the structure of the NJ network (Figure 5). In the NJ network, all the populations from Yili Valley clustered into a clade (Yili Valley clade), sister to the other clade containing all the populations from western Ordos Plateau (western Ordos clade). Yili Valley clade consists of two inner clade: the first inner clade contains populations BSD, BL, and LK, and the second clade contains the remaining populations. In the first inner clade, population BSD are separate from populations BL and LK. In western Ordos clade, populations MX, QLG, and QLS from the north of the plateau cluster together, drifting apart from populations HN, KBQ, and QPJ, from the south of the plateau.

## Discussion

### Genetic divergence between Yili Valley and western Ordos Plateau

Both the nuclear and chloroplast phylogenetic analyses showed two distinct lineages in *Helianthemum*, distributed in Yili Valley and western Ordos Plateau (Figures 1, 2). NJ network and PCO plots also indicated the similar result (Figures 4, 5). AMOVA analysis and Mantel test both showed a high level of genetic divergence between the two regions. In addition, ploidy in *H. songaricum* is 2X while in *H. ordosicum* is 4X. Wiley (1978) stated “A single lineage of ancestral descendant populations of organisms which maintains its identity from other such lineages and which has its own evolutionary tendencies and historical fates, should be defined as species status.” The two single lineages in *Helianthemum* in northwestern China clarified the taxonomic confusion of the genus, supporting that populations in western Ordos Plateau should be given species rank, *H. ordosicum*, supposed by Zhao et al. (2000).

In early Tertiary, the terrain and climate of northwestern China were very different from the arid and mountainous conditions of today. Some species of ancient Mediterranea flora, such as *Helianthemum*, spread along the relic of ancient Mediterranea distribution, across the Hexi Corridor, arrived to Alxa desert (Czenda, 1977; Liu, 1995). In the late Tertiary, uplifting of the northern Tibetan Plateau caused extensive aridification in northwestern China (Zheng et al., 2003; Sun et al., 2008). During the Quaternary, glaciation began to developed in the Northern Hemisphere, and the colder climate reached its maximum at about 0.8–0.6 Ma (Williams et al., 1993). Due to the dramatic climate change, many plant species of deserts in northwestern China gradually became extinct (Liu, 1995). This climatic history suggests that ancestral *Helianthemum* distributed in Hexi Corridor, a passage connecting Yili Valley with western Ordos Plateau, became extinct leaving those distributed in Yili Valley and western Ordos Plateau as relics. This hypothesis is supported by the haplotype relationships shown in the median-joining network (Figure 2). The putative extinction of *Helianthemum* along this corridor limited gene flow between the two regions. In addition to restricted gene flow, the climate in Yili Valley likely differentiated from that of the western Ordos Plateau. Because of the Tianshan Mountains, the Yili Valley has a Central Asia climate (Liu, 1995) with hot- dry summers, and mild-humid springs and winters (Shi et al., 2005). However, climate in western Ordos Plateau is typically drier throughout the year (Walker, 1974). *Helianthemum* in these two regions have inhabited distinct habitats for several millennia, harboring unique populations.

### Genetic diversity and genetic structure

Total genetic diversity of the two species are both moderate (*H*. *songaricum*: h_T_ = 0.583; *H. ordosicum*: h_T_ = 0.634), compared with other desert plants, such as *Ammopiptanthus mongolicus* (h_T_ = 0.434), *A. nunas* (h_T_ = 0.041) (Su et al., 2016), *Reaumuria soongorica* (h_T_ = 0.312) (Qian et al., 2008), and are both higher than that in the previous phylogeography study of *Helianthemum* (*H. songaricum*, h_T_ = 0.162; *H. ordosicum*, h_T_ = 0.566), using two chloroplast intergenic spacers (Su et al., 2011). The inconsistency is due to higher polymorphism in the three cpSSRs than the two chloroplast spacers.

The cpSSRs data showed signs for genetic divergence in both *H. songaricum* and *H. ordosicum*. AMOVA analysis demonstrated significant genetic divergence among populations in both *H. songaricum* and *H. ordosicum*. The genetic divergence in the two species were also supported by NJ network and PCO analysis. As shown by the NJ network and PCO plots (Figures 4, 5), populations BL, LK, and BSD clustered together, apparently separated from the other populations in Yili Valley; populations HN, KBQ, and QPJ clustered together, apparently separated from the other populations in western Ordos Plateau. The significant genetic divergence among populations in the two species might be attributed to several factors. First, the seed viability is poor. In a *Helianthemum* flower, most ovules are unfertilized, or fertilized but with abnormal development, usually leaving only 1-3 well-developed seeds. Thus, seed production is very low (Ma et al., 2007). In addition, the seed requires a dormancy period, and with the poor water translocation due to the hard testa (Cao et al., 2000), the germination is very low (Ma et al., 2007). Second, habitats of the two species are both highly fragmented. In Yili Valley, there are several mountain ranges that subdivide *Helianthemum* habitat into five valleys (Zhang, 2006). The collected sites of *H. songaricum* are located in different secondary valleys. Similar in western Ordos Plateau, collection sites of *H. ordosicum* located in different valleys, along the Table Mountains. Within each species, the numerous geographic barriers isolate the populations, obstructing gene flow among them, and consequently likely decreasing genetic diversity and increasing the genetic divergence. Third, a reduced population size in the two species might also affect the population structure by increased selfing, mating among related individuals, and genetic drift. Based on congeners (Rodríguez-pérez, 2005; Aragón and Escudero, 2008), *H. songaricum* is likely an outcrosser but also self-compatible. Increased selfing or mating among related individuals in small population would reduce heterozygosity (Schaal and Leverich, 1996), and increased genetic drift would fix alleles randomly (Lynch et al., 1995), resulting in an alteration of population allele composition, inducing the population as an unique genetic sector (Gaudeul et al., 2000). The pattern of single haplotypes found in nearly all the populations in the Yili Valley suggests selfing is a fixture of this region. Compared to *H. songaricum, H. ordosicum* populations have greater population diversity (Table 3), and typically multiple haplotypes per population (Figure 1). These contrasting patterns suggest a greater degree of selfing or inbreeding in *H. songaricum* that could be caused by barriers to gene flow, differences in the abundance of pollinators or adaptation to a selfing life history.

### Implications for conservation in *Helianthemum*

Habitat fragmentation is a significant threat to the survival of plant species in many terrestrial ecosystems (Young et al., 1996). Our data observed low genetic diversity in isolated small populations in *Helianthemum* in northwestern China. Low genetic diversity can reduce population fitness and viability, weakening the population's ability to respond to changing selection pressures, increasing the extinction vulnerability (Young et al., 1996). We suggest an effective conservation management program incorporated our genetic analysis in following manners: (i) Significant genetic divergence showed by both nuclear and chloroplast data indicates two single evolutionary lineages in *Helianthemum* in northwestern China. The two species should be treated, respectively, when performed a management strategy. (ii) For in situ conservation, all the natural habitat of *Helianthemum* populaton should be preserved by local governments. Nature reserves for *H. ordosicum* have been set up in western Ordos Plateau. However, in Yili Valley, conservation of *H. songaricum* has not been given enough attention, and we propose nature reserves for *H. songaricum* should be set up at once. In addition, for serious fragmentation in both species, extinct populations should be reestablished to connect remnant populations in each species, using progenies from populations with nearest geographic distance. Also, population sizes should be augmented by transplanting progenies propagated from original populations. (iii) For both species, ex situ conservation site should be established first. Seeds collection should capture all detected genetic variations to represent the genetic diversity of each species in maximum, avoiding artificially induced bottlenecks (Maunder et al., 2001). Because of genetic uniqueness of populations BSD, BL, and LK in *H. songaricum*, seeds collections in these populations should be deposited as separate stocks. Meanwhile, seedlings of each species should be cultured for their future restoration. In *H. songaricum*, crossing individuals from unique populations (BSD, BL, and LK) and the rest of the populations should be tested ex situ to prevent potential outcrossing depression.

## Author contributions

Conceived and designed the experiments: ZS, BR. Performed the experiments: ZS. Analyzed the data: ZS, BR, LZ, and XJ. Contributed reagents/materials/analysis tools: ZS. Wrote the paper: ZS.

### Conflict of interest statement

The authors declare that the research was conducted in the absence of any commercial or financial relationships that could be construed as a potential conflict of interest.
